# Right ventricular dysfunction in left ventricular assist device candidates: is it time to change our prospective?

**DOI:** 10.1007/s10741-024-10387-w

**Published:** 2024-02-08

**Authors:** Carlotta Sciaccaluga, Maria Cristina Procopio, Luciano Potena, Marco Masetti, Sonia Bernazzali, Massimo Maccherini, Federico Landra, Francesca Maria Righini, Matteo Cameli, Serafina Valente

**Affiliations:** 1 Department of Medical Biotechnologies, Division of Cardiology, University of Siena, Siena, Italy; 2 Department of Clinical and Experimental Medicine, University of Messina, Messina, Italy; 3 Heart Failure and Transplant Unit, IRCCS Azienda Ospedaliero-Universitaria di Bologna, Bologna, Italy; 4 Department of Cardiac Surgery, University of Siena, Siena, Italy

**Keywords:** LVAD, Right ventricular failure, Right ventricular dysfunction

## Abstract

The use of left ventricular assist devices (LVAD) has significantly increased in the last years, trying to offer a therapeutic alternative to heart transplantation, in light also to the significant heart donor shortage compared to the growing advanced heart failure population. Despite technological improvements in the devices, LVAD-related mortality is still fairly high, with right heart failure being one of the predominant predictors. Therefore, many efforts have been made toward a thorough right ventricular (RV) evaluation prior to LVAD implant, considering clinical, laboratory, echocardiographic, and invasive hemodynamic parameters. However, there is high heterogeneity regarding both which predictor is the strongest as well as the relative cut-off values, and a consensus has not been reached yet, increasing the risk of facing patients in which the distinction between good or poor RV function cannot be surely reached. In parallel, due to technological development and availability of mechanical circulatory support of the RV, LVADs are being considered even in patients with suboptimal RV function. The aim of our review is to analyze the current evidence regarding the role of RV function prior to LVAD and its evaluation, pointing out the extreme variability in parameters that are currently assessed and future prospective regarding new diagnostic tools. Finally, we attempt to gather the available information on the therapeutic strategies to use in the peri-operative phase, in order to reduce the incidence of RV failure, especially in patients in which the preoperative evaluation highlighted some conflicting results with regard to ventricular function.

## Introduction

Despite substantial improvements in medical and mechanical management, patients with advanced heart failure are still suffering from a high mortality rate [1]. The only long-term treatment strategies for these patients are heart transplantation and left ventricular assist devices (LVAD). In those ineligibles for transplantation or in patients waiting for transplant, LVAD represents the only durable option, even though long-term outcomes post-LVAD are still lower compared to heart transplant. Indeed, the recently published results of the extended follow-up of the MOMENTUM 3 trial showed that 5-year survival after LVAD is about 58.4%, mainly limited by related complications, particularly right heart failure (RHF) [2]. Early post-implant RHF is estimated to occur in as high as 35% of LVAD patients [3, 4] while late RHF in approximately 10% of them [5], and their occurrence is associated with a 3.8-fold increase in mortality [6]. Adequate RV function is a requirement of fundamental importance for patients undergoing LVAD implant. In fact, it is essential to permit sufficient anterograde flow for proper device functioning. Therefore, a careful preoperative and intraoperative assessment of the RV is crucial in candidates for LVAD. Plentiful studies have investigated various parameters potentially correlated with the onset of RHF post-implant, although no univocal score or cut-off is available [7, 8]. That being said, in clinical practice, it is not rare to face patients with so-called “borderline” RV function. There is no univocal definition of borderline RV function, but it is generally applied to patients in whom even a thorough evaluation highlights conflicting parameters of RV function or opposing RHF predictive scores. For instance, RV borderline function could be attributed to a patient that shows echocardiographic parameters below the prognostic cut-off values for RHF post-LVAD but good hemodynamic indexes of RV function. These patients impose a particularly though clinical decision to proceed with LVAD implant due to the uncertainty of the risk of RV dysfunction after the implant. However, in recent years, growing evidence has been pointing to expand LVAD implant even to patients with suboptimal RV function, especially in light of technological improvement in RV mechanical support [9, 10].

The aim of this review is to gather the available evidence on RHF in LVAD candidates which is a starting point to face and possibly overcome the aforementioned challenges. In particular, the focus of this review is LVAD candidates with borderline RV function. Starting from physiopathology of post-implant RHF, future perspective to evaluate RV contractile reserve as well as therapeutic options to support RV function before and after LVAD implant are presented.

## Definition and physiopathology of right heart failure

RHF is a clinical syndrome with signs and symptoms of HF resulting from RV dysfunction, defined as evidence of abnormal RV structure or function [11]. In the most updated definition of adverse events after mechanical circulatory support (MCS) by Mechanical Circulatory Support Academic Research Consortium, RHF is divided in early acute, early post-implant and late RHF. Early acute RHF occurs during surgery and requires implantation of a temporary or durable RVAD [12]. Early post-implant RHF occurs within 30 days from surgery and either requires implantation of an RVAD or inotropes/vasopressors/inhaled nitric oxide for more than 14 days following LVAD implantation. Need for implantation of an RVAD (including ECMO) greater than 30 days after an LVAD implantation or hospitalization that occurs more than 30 days post-implant and which requires intravenous diuretics or inotropic support for at least 72 h eventually defines late RHF. The grading system of RHF in mild, moderate and severe has been abandoned [12]. Table 1 summarizes the main definitions of RHF used in clinical trials.

**Table 1:** Most commonly used definitions of right ventricular failure

	**Definition of Right Heart Failure**
**MCS-ARC **[12]	Presence of at least two of the following clinical findings: • Ascites • Functionally limiting peripheral edema (> 2 +) • Elevated estimated jugular venous pressure at least halfway up the neck in an upright patient • Elevated measured central venous pressure or right atrial pressure (≥ 16 mm Hg) Or at least one of the following manifestations: • Renal failure with serum creatinine > 2 × baseline values • Liver injury with an elevation of at least 2 × upper limit normal in AST/ALT or total bilirubin > 2.0 • SVO2 < 50% • Cardiac index < 2.2 L/min/m^2^ • Reduction in pump flow of > 30% from the previous baseline in the absence of mechanical causes such as cardiac tamponade or tension pneumothorax • Elevated lactate > 3.0 mmol/liter
**INTERMACS **[53]	• Manifestations of elevated CVP^a^ • Documented CVP or RAP > 16 mmHg Severity graded from mild to severe and severe-acute
**EUROMACS RHF risk score **[10]	• Need for RV circulatory support or • Need for continuous inotropic support for ≥ 14 days or • Need for nitric oxide ventilation for ≥ 48 h
**ASE and EACVI **[54]	• RV-FAC < 35% • TAPSE < 17 mm • Tricuspid lateral annular systolic velocity (*S*′) < 9.5 cm/sec • RIMP > 0.43 by pulsed Doppler or > 0.54 by tissue Doppler

*ALT* alanine transaminase, *ASE* American Society of Echocardiography, *AST* aspartate transaminase, *CVP* central venous pressure, *MCS-ARC* mechanical circulatory support-Academic Research Consortium, *EACVI* European Association of Cardiovascular Imaging, *RAP* right atrial pressure, *RHF* right heart failure, *RIMP* right ventricular index of myocardial perfusion, *RVFAC* right ventricular fractional area change, *TAPSE* tricuspid annular plane systolic excursion

^a^Clinical findings of peripheral edema (≥ 2 + either new or unresolved) or presence of ascites or palpable hepatomegaly on physical examination (unmistakable abdominal contour) or by diagnostic imaging

Many factors are implicated in the complex physiopathology of RHF. First of all, it is noteworthy that RV systolic function is mainly carried by longitudinal fibers shortening, which is substantially affected by pericardiotomy [13]. Moreover, the RV free wall, which accounts for the largest contribute to systolic function, is more susceptible to ischemic injury induced by prolonged cardio-pulmonary bypass and bleeding. Regarding hemodynamic changes induced by LVAD, it provides a reduction in pulmonary capillary wedge pressure (PCWP) and so in RV afterload. However, on the other hand, an extreme LV decompression may result in a leftward shifting of the interventricular septum (particularly of the apical segment), which usually accounts for 20–40% of RV systolic function, altering RV shape and size, thereby affecting its contractility. In addition, tricuspid regurgitation might be worsened as well. Finally, an improved LVAD-induced cardiac output together with perioperative transfusions and intravenous fluid administration, increase venous return, overloading right-sided chambers.

Summarizing, once the device is activated the interventricular septum tends to shift towards the left ventricle as it unloads, the systemic venous return is increased and tricuspid regurgitation might be augmented especially if there is a dilatation of the RV or a primary valvulopathy, factors that could lead to RHF especially in patients with preexisting RV dysfunction. Taking that into account, if on one hand it is essential to optimize the speed in order to minimize these effects, on the other it is of pivotal importance an adequate preoperative selection based on RV function.

Regarding late RHF, while having an identical clinical presentation as compared to early RHF, it shows a different physiopathology, which is equally multifactorial. In such case, three possible conditions may be involved in disease development (not considering drugs discontinuation): RV failure, aortic insufficiency, and ventricular arrhythmias [14]. In addition to previously explained causes of RV failure, intrinsic myocardial disease progression plays a pivotal role. For what it concerns aortic insufficiency, it is well known that LVAD itself may cause progressive valve degeneration with increased degree of regurgitation, which leads to left ventricular end-diastolic pressure and RV afterload increase. Finally, ventricular arrhythmias may result from all these conditions, further worsening the whole picture in a vicious cycle, affecting RV systolic function and AV opening with consequent increase in aortic regurgitation.

## Preoperative risk assessment of right heart failure after LVAD implant

The occurrence of RHF after LVAD is still associated with higher rates of HF hospitalization as well as higher mortality [15]. Despite its prognostic impact, its prevalence is still significant, ranging from 9 to 40% depending on the definition used [15]. Therefore, one of the main objectives of an LVAD candidate’s evaluation is to effectively assess the risk of developing RHF, since when the severity of RV dysfunction is underestimated before and during LVAD placement, RV support might be delayed, with a negative impact on outcome.

Yet, literature does not support a consensus of which preoperative parameters can be used to optimal patients’ selection, even though several studies have detected many preoperative predictors of RHF post-LVAD implantation. Throughout the years, several indexes have been investigated in this setting, including clinical, laboratory, echocardiographic, and hemodynamic parameters. Table 2 shows the most significant predictors and their cut-off variability according to different studies, which contributes to the great heterogeneity in the risk assessment of RHF post-LVAD. RV dysfunction is generally linked to systemic venous congestion which greatly affects both liver and kidneys, resulting in an impairment of their functions. In facts, evidence points to a strong predictive power of renal parameters, for instance a value of serum creatinine above 1.9–2.3 mg/dl suggests a greater risk of RHF [16, 17] as well as blood urea nitrogen, with values above 39–44.5 mg/dl [18, 19]. Furthermore, the review and meta-analysis by Bellavia et al. showed that, among biochemical parameters, high NT-proBNP levels, INR and white blood count are correlated with RHF [20]. Therefore, a patient with advanced heart failure in an optimized volume status that presents elevated transaminase levels as well as bilirubin and INR, should be careful evaluated before LVAD since the chances of a clinical or subclinical RV dysfunction are high.

**Table 2:** Predictors of right ventricular failure post-left ventricular assist device implant

**Predictors**	**RHF high-risk value**
**Laboratoristic parameters**	
• Serum creatinine	• > 1.9 mg/dl [15]
	> 2.3 mg/dl [17]
• Blood urea nitrogen	• > 39 mg/dl [18]
• NT-proBNP	> 44.5 mg/dl [19]
• WBC	• per pg/ml increase [20]
• INR	• per × 10^3^ UL increase [21]
	• per unit increase [20]
**Echocardiographic parameters**	
• TAPSE	• < 12.5 mm [21]
	• < 7.5 mm [55]
• RVFAC	• < 31% [21]
• fwRVLS	• > − 5.64% [56]
	• > − 7.9% ± 1.2 9[23]
	• > − 9.6 [24]
	• > − 11% [26]
	• > − 15.5% [27]
	• > 0.72 [56]
** • ***R*/*L* ratio	
**Hemodynamics parameters**	
• PAPi	• < 2 [44, 57]
• CVP/PCWP	• > 0.63 [18]
• RVSWI	• < 400 mmHg × ml/m^2^ [27]
	• ≤ 250 mmHg × ml/m^2^ [16]
	• < 300 mmHg × ml/m^2^ [58, 59]
• CI	• < 2.2 l/min/m^2^ [16]

*CI* cardiac index, *CVP* centrale venous pressure, *FWRVLS* free wall right ventricular longitudinal strain, *INR* international normalized ratio, *PAPi* pulmonary artery pulsatility index, *PCWP* pulmonary capillary wedge pressure, *RHFAC* right ventricular fractional area change, *RVSWI* right ventricular stroke work index, *TAPSE* tricuspid annular plane systolic excursion, *WBC* white blood cells

Transthoracic echocardiography (TTE) also plays a key role, since it is an easily accessible and noninvasive tool for evaluating RV anatomy and function. However, there is not a univocal consensus regarding neither which echocardiographic parameter nor which cut-off value is the best predictor of RHF post-LVAD. For instance, the first available evidence pointed toward the prognostic role of tricuspid annulus plane systolic excursion (TAPSE), which measures RV longitudinal shortening. Also right ventricular fractional area change (RVFAC) plays a significant role and a value below 31% was associated with a high risk of developing RHF post-LVAD implantation [21], even though Bellavia et al. highlighted its low effect size in predicting RHF [7]. In recent years, the measurement of RV free wall longitudinal strain (fwRVLS) has been applied to this setting of patients, showing promising results, overcoming most of the limitations of the aforementioned RV parameters. Indeed, several studies showed the incremental prognostic value of fwRVLS compared to other echocardiographic as well as laboratory and hemodynamic indexes. Stricagnoli et al. showed that RHF was associated with reduced free wall right ventricular longitudinal strain (fw-RVLS) (− 7.9 ± 1.29 vs. − 16.14 ± 5.83, *p* < 0.009), which was superior to other echocardiographic determinants of RHF [22]. Andrew et al. demonstrated that a peak RV strain cut-off of − 9.6% predicted RHF with 76% specificity and 68% sensitivity [23] highlighting however the importance of incorporating multiple factors into the risk assessment. Fw-RVLS was also incorporated into the SIENA protocol by Cameli et al., with a cut-off value of − 11% for RHF prediction [24]. However, there is still high variability between different studies, which explain the reason why this parameter is resulted to be only marginally significant when comparing RHF vs. No-RHF patients [4]. In light of the current evidence, the best echocardiographic predictor of RHF post-LVAD implant seems to remain a purely qualitative assessment of RV function [7, 20], thus prompting an implementation in studies testing the value of new predictors in larger cohort of patients [25].

Hemodynamic assessment through right heart catheterization is considered a fundamental tool to assess RV function, although this evaluation can be affected by many factors, as inotropic support and patient’s volume status. The pulmonary artery pulsatility index (PAPi) is considered a robust, independent predictor of RHF or the need for RVAD device post-LVAD implantation at the cut-off of 1.85 with a sensibility of 94% and a specificity of 81% [26], which is currently one of the mainly used parameter in clinical practice. Another useful invasive parameter is central venous pressure/capillary pulmonary wedge pressure (CVP/CPWP) ratio, that when above 0.63 emerged as independently predictive risk factor of RHF [18]. The higher this ratio is, the higher is the likelihood of RV dysfunction since CVP is disproportionately high compared to CPWP. However, caution should be exercised for high values of CVP/CPWP ratio because, on the one hand, elevated CVP is suggestive of RHF but, and on the other hand, it could be correlated to volume overload. Hence, high-dose diuretic therapy, often associated with inotropic support, and ultrafiltration, in case of diuretics’ resistance, is advisable to contrast volume overload. RV stroke work index (RVSWI) is considered one of the most significant parameters that need to be evaluated when assessing the risk of post-LVAD RHF. Indeed, RVSWI below 400 mmHg × ml/m^2^ is an independent predictor of RHF after LVAD implantation [27] and a value below 250 mmHg ml/m^2^ is independently associated with the need for BiVAD placement [14]. Dandel et al. identified CVP and mean pulmonary arterial pressure as independent preoperative predictors of postoperative RHF [28]. Finally, Bellavia et al. showed that among hemodynamics variables, high CVP, low RVSWI, and mean arterial pressure are the ones most strongly associated with RHF [4].

## Predictive risk score of right heart failure

Despite the existence of aforementioned preoperative predictors of RHF, RV behavior after LVAD placement is difficult to predict. With the purpose to increase the ability to correctly stratify the risk of RHF post-LVAD, several RHF risk scores have been developed, which combine clinical, laboratory, and hemodynamic measurements, proving to be additional tools for cardiologists to guide adequate patients’ selection. Table 3 summarizes the main predictive scores of RHF after LVAD.

**Table 3:** Principal predictive scores of right ventricular failure post-left ventricular assist device implant

**Scoring system**	**Predictors**	**Cut-off value**	**Points**	**High-risk of RHF**
Matthews’ score (Michigan score) [17]	• Vasopressors’ need • AST • Bilirubin • Creatinine (or RRT)	• Yes/no • ≥ 80 UI/l • ≥ 2 mg/dl • ≥ 2.3 mg/dl	• 4 • 2 • 2.5 • 3	> 5.5 points
Fitzpatrick’s score (Pennsylvania score) [16]	• CI • RVSWI • Creatinine • Pre cardiac surgery • RVD • SBP	• ≤ 2.2 L/min/m^2^ • ≤ 250 mmHg ml/m^2^ • ≥ 1.9 mg/dl • Yes/no • Yes/no • ≤ 96 mmHg	• 0/1 • 0/1 • 0/1 • 0/1 • 0/1 • 0/1	> 65 points
Drakos’ score (Utah score)[60]	• LVAD as DT • IABP • PVR • Inotrope dependency • Obesity • ACEi or ARB • β blockers	• Yes/no • Yes/no • ≤ 1.7 wood units • Yes/no • Yes/no • Yes/no • Yes/no	• 3.5 • 4 • 4 • 2.5 • 2 • − 2.5 • 2	> 12.5 points
Pittsburgh decision tree score [61]	• TPG • Age • RAP • INR • HR • WBC • ALT • Number of inotropes	• > 7 mmHg • > 59 years • > 18 mmHg	The presence of all of them increased risk of RVAD If RAP < 18 mmHg, consider, HR, WBC, and inotropes	The combination of the three elements
CRITT score [62]	• PVC • Severe RVD • Preoperative Intubation • Severe tricuspid regurgitation • Tachycardia	• > 15 mmHg • Yes/no • Yes/no • Yes/no • Yes/no	• 0/1 • 0/1 • 0/1 • 0/1 • 0/1	≥ 4 points
ARVEDE score [63]	• INTERMACS I • RVEDD • *E*_*m*_/*S*_*lat*_	• Yes/no • ≥ 50 mm • ≥ 18.5	• 1.5 • 2 • 3	> 3 points
MCSRN score [64]	• HR • Albumin • BUN • WBC • CI • TR severity	4.2944 * log(HR) − 4.4917 * log(Albumin) + 1.2029 * log(BUN) + 1.0599 * log(WBC) − 1.0364 * log(CI) + 0.8213 * numeric TR severity		> 22
HMRS [65]	• age • Albumin • Creatinine • INR • Center LVAD volume	(0.0274 × age [in years]) − (0.723 × albumin [g/dl]) + (0.74 × creatinine [mg/dl]) + (1.136 × INR) + (0.807 × center LVAD volume *) *Enter value of 1 if total center LVAD < 15		> 2.48
EUROMACS score [20]	• Severe RVD • PVC/PCWP ratio • INTERMACS • Inotropes • Hb	• Yes/no • ≥ 0.54 • 1–3 levels • ≥ 3 • ≤ 10	• 2 • 2 • 2 • 2.5 • 1	> 4 points
ALMA score [58]	• DT intention • PAPi • RVSWI • RV/LV ratio • MELD- XI score	• Yes/no • < 2 • < 300 mmHg/ml/m^2^ • > 0.75 • > 17	• 0/1 • 1 • 1 • 1 • 1	2–3 points (57.1% risk) 4–5 points (100% risk)
Siena score [24]	• FWRVLS • RHFAC • 3D RVEF • RVSI	• > − 11% • < 35% • < 35% • > 0.5	• 1 • 1 • 1 • 1	> 1 point

*ACEi* angiotensin-converting enzyme inhibitors, *ALT* alanina aminotransferase, *ARB* angiotensin receptor blockers, *AST* aspartate aminotransferase, *BUN* blood urea nitrogen, *CI* cardiac index, *DT* destination therapy, *FWRVLS* free wall right ventricular longitudinal strain, *Hb* haemoglobin, *HR* heart rate, *IABP* intra-aortic balloon pump, *LVAD* left ventricular assist device, *MELD-XI score* Model For End-Stage Liver Disease Excluding International Normalized Ratio, *PAPi* pulmonary artery pulsatility index, *PCWP* pulmonary capillary wedge pressure, *PVC* central venous pressure, *PVR* pulmonary vascular resistance, *RAP* right atrial pressure, *RRT* renal replacement therapy, *RV* right ventricular, *RV/LV ratio* right to left ventricular end diastolic diameter ratio, *RVEDD* right ventricular end diastolic diameter, *RHFAC* right ventricular fractional area change, *RVSI* right ventricular sphericity index, *RVSWI* right ventricular stroke work index, *SBP* systolic blood pressure, *TPG* transpulmonary gradient, *TR* tricuspid regurgitation, *WBC* white blood count, *3D RVEF* 3-dimensional right ventricular ejection fraction

Several factors should be considered for the limitations of these scoring models. First, most of them have been tested retrospectively and on a limited population. Second, some of these scoring systems are developed in both pulsatile-flow and continuous-flow LVAD era, thus contributing to an inability to reproduce the same results in patients implanted with the contemporary continuous-flow LVADs. Third, during these studies, the definition of RHF used was often different, also because of the lack of universal definition of RV dysfunction; for instance, some studies defined RHF post-LVAD only as receiving right-sided circulatory support, while other studies defined RHF also as continuous inotropic support or the use of nitric oxide. In the latter case, it is also important determinate the specific used inotropic agent as well as the infusion duration, which may vary across different operators and different centers, thus resulting in further selection bias. Fourth, thanks to the progresses in echocardiographic techniques, as introduction of speckle-tracking echocardiography, many measurements now commonly used in echocardiographic laboratories and considered independent predictors, as fw-RVLS, are not included in these scoring models. New scoring models developed with multicenter and prospective studies using similar inclusion criteria and possibly a common definition of RV dysfunction are necessary. In fact, a universal definition of RV dysfunction should be advocated, in order to avoid study variability and inconsistencies in study outcomes and better selection of candidates for RV support.

That will help to optimize clinical decision-making for the prediction of RV performance in patients addressed to LVAD implantation, creating a more homogeneous patient population coming from different centers.

### Dynamic assessment of right ventricular function

**Fig. 1:**
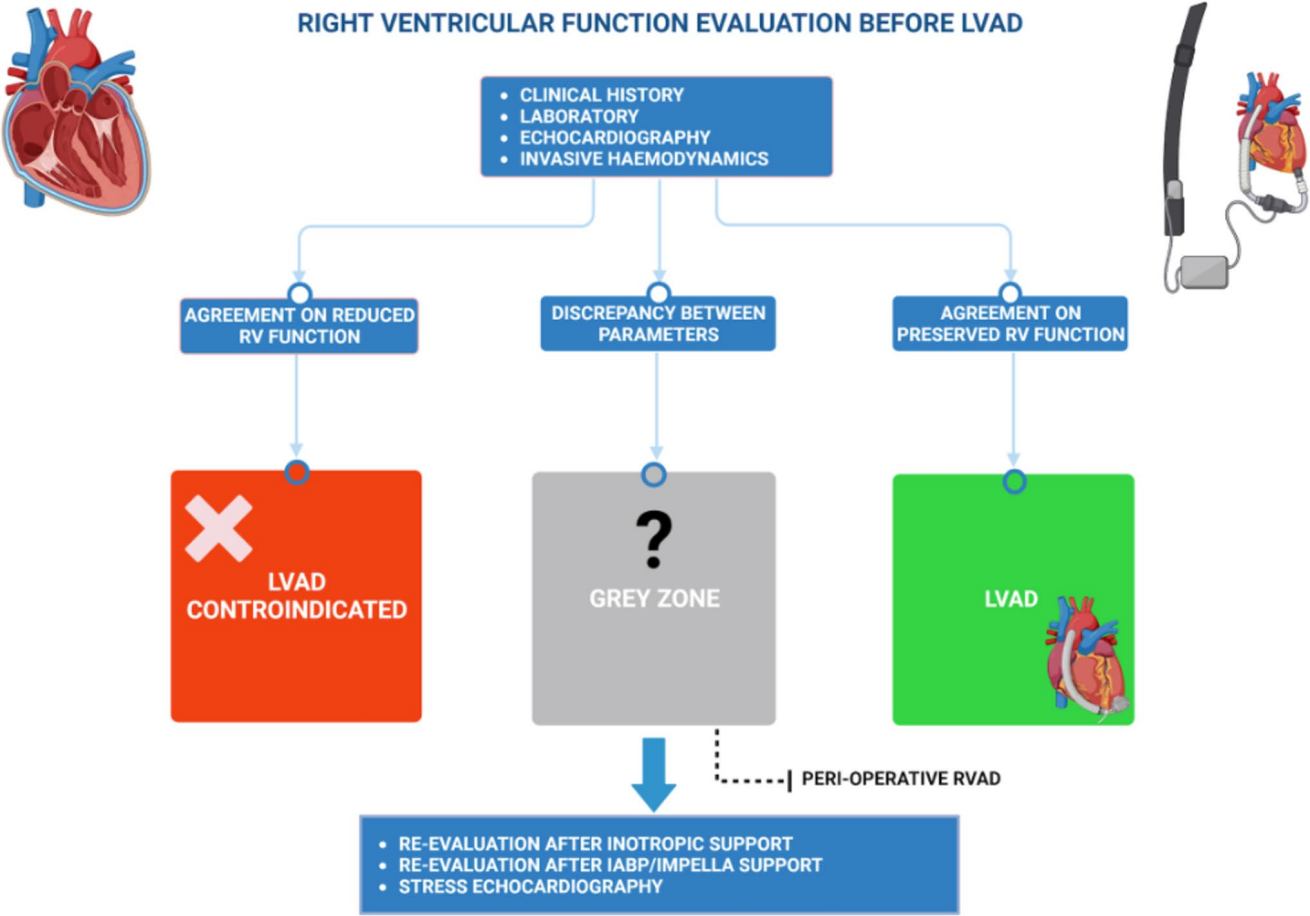
Preoperative evaluation of right ventricular function before left ventricular assist device implant. The evaluation of right ventricular (RV) function before left ventricular assist device (LVAD) is essential in order to assess the individual risk of developing right heart failure after implant and it comprises clinical history, laboratory, echocardiography, and invasive hemodynamics as well. However, due to the high heterogeneity in the available parameters, there is a significant proportion of patients in which it is not possible to clearly distinguish an acceptable RV function and a poor RV function, which contraindicates LVAD implant. In these patients, new diagnostic tools are advocated in order to better stratify the peri-operative risk of RV failure, such as re-evaluation after inotropes, IABP/Impella support or RV contractile reserve through stress echocardiography. Finally, in these patients, the use of peri-operative right ventricular assist device (RVAD) could represent a valid solution in order to prevent rather than treat RV failure post-implant

In light of the proposed evidence, it is not surprising to encounter conflicting results regarding the risk of developing RHF post-LVAD in the same patient. Therefore, if on one hand therapeutic strategies have been implemented in the latest years to support suboptimal RV in the peri-operative period, on the other it would be useful to try to find other tools to better estimate RV contractile function (Fig. 1). For instance, echocardiographic resting measurements may not be enough to predict RV functional adaptation to increased flow output secondary to LVAD-induced LV unloading. Hence, attention has been focusing on stress doppler echocardiography which potentially enables to assess RV contractile reserve, increasing the preoperative predictive ability of RHF.

From a physiologic point of view, ventricular contractile (or inotropic) reserve is defined as a systolic ventricular response to exercise or pharmacological stimulation through a change in ejection fraction or stroke volume [29]. Contractile reserve depends on several factors, such as ventricular contractility, myocardial interstitial fibrosis, ventricular remodeling, ventricular afterload, myocardial perfusion, and response to neurohormonal activation [29]. In contrast to the established prognostic role of stress-echocardiography-derived LV contractile reserve in several contexts [30–32], several measures of RV function have been proposed to evaluate RV contractile reserve, as TAPSE, tricuspid *S*′, and RVFAC but they are not validated yet. Although there are no investigations on LVAD candidates, several studies on patients with pulmonary hypertension might provide some clues for RV contractile reserve evaluation in candidates for LVAD implantation, also because many of these patients often enough have a variable degree of pulmonary hypertension. In patients with pulmonary artery hypertension, the primary mechanism of RV adaptation to raised afterload secondary to increased pulmonary vascular resistance and decreased pulmonary vascular compliance is an increase in contractility up to 4–5fold to preserve stroke volume. The ability of RV to adapt its contractility to increased afterload is called coupling between RV and pulmonary circulation. Sharma et al. identified two echocardiographic indices of RV contractile reserve, TAPSE, and tricuspid *S*′, in patients with pulmonary arterial hypertension compared with control group by using both treadmill cardiopulmonary exercise testing and dobutamine stress testing [33]. They found that even in patients with pulmonary arterial hypertension where parameters were within the normal range at baseline, contractile reserve remained significantly reduced compared to healthy controls, suggesting that stress testing may potentially unmask latent RV dysfunction that is not evident at rest [33]. One of the first studies was conducted by Grunig et al. who used pulmonary artery systolic pressure (PASP) as an indirect measure of RV contractile reserve and noted that an exercise-induced PASP increase of 30 mmHg by stress echocardiography in patients with pulmonary hypertension results from an increase of cardiac output, demonstrating RV ability to improve its stroke volume during stressing events [34]. However, after LVAD implant, the RV has to face an increased preload rather than an increased afterload, therefore prognostic stress-echocardiographic parameters should be investigated specifically in this setting.

Furthermore, although the benefits of levosimendan on the left ventricle are well recognized, limited data are available on its utilization in RHF. The assessment of RV function before and after infusion of levosimendan showed interesting insights. Parissis et al. evaluated RV function in a cohort of 54 consecutive patients with advanced heart failure, who were randomized to receive either levosimendan or placebo; they demonstrated a statistically significant increase in TDI maximal systolic tricuspid annular velocity (8.2 ± 3.2 vs 9.0 ± 3.0 cm/s, *p* < 0.03), as well as a significant reduction in PASP (54 ± 11 vs 43 ± 11 mm Hg, *p* < 0.01) in patients treated with levosimendan [35]. Another study was performed on 59 patients with acute decompensated heart failure, of whom 30 patients were randomized to receive levosimendan and 29 patients were randomized to receive placebo. That study showed an improvement of RV echocardiographic-derived parameters, such as TDI tricuspid *S*′ velocity, RVFAC, and TAPSE after levosimendan infusion [36]. It would be useful to study how levosimendan could affect advanced echocardiographic parameters of RV function, such as longitudinal strain and 3D-ejection fraction, in patients awaiting LVAD and if it has an impact on RHF. Besides echocardiography, Sponga et al. assessed the effects of preoperative levosimendan on hemodynamic parameters in 21 LVAD patients with preoperative borderline RV function [37]. Borderline RV function was considered when at least one the following criteria was met: RV end-diastolic volume > 35 mm, RV ejection fraction < 30%, tricuspid regurgitation > II grade, short/long axis ratio of right ventricle > 0.6, and pulmonary pressure > 35 mmHg. After 48 h of levosimendan infusion, the cardiac index increased significantly by 21% (*p* = 0.014), pulmonary pressure decreased by 12% (*p* = 0.003), whereas PCWP and CVP decreased by 15% (*p* = 0.028 and *p* = 0.016), respectively [37]. That being said, to date, despite these interesting insights, there is a lack of conclusive evidence regarding the association between levosimendan infusion and post-operative outcomes, including RHF [38]. Despite the fact that no study has proven its efficacy on preventing RHF post-LVAD, its use before LVAD could provide prognostic information through the use of natriuretic peptides. In fact, Sponga et al. showed that a reduction of NT-proBNP below 25% from baseline values (before levosimendan infusion) is a strong predictor of mortality (100% sensitivity and 70% specificity) [37]. Therefore, levosimendan could be useful not only for preoperative optimization but also for a better risk stratification, allowing to plan preemptive peri-operative strategies.

Finally, in LVAD candidates with advanced heart failure that necessitate short-term mechanical support, the use of aortic counterpulsation and mostly Impella device might provide additional information on how the RV responses to an increased preload, similarly to the one induced by a long-term LVAD, even though data are still lacking.

## Therapeutic strategies to prevent right heart failure

Various therapies might be endorsed in case of RHF, starting from hemodynamic optimization, inotropic support, inhaled nitric oxide to temporary mechanical support. However, in those patients in whom the estimated likelihood of RHF post-LVAD is high for patients with preoperative suboptimal RV function, it is useful to implement strategies in the peri-operative period in order to avoid the onset of LVAD-related RHF rather than treating it once it has occurred. The clue aspects that have to be analyzed before LVAD implant in patients with borderline RV function are defining the individual risk of developing RHF according to the available scores, the patient’s RV contractile function and lastly the patient’s indication to LVAD. Once these aspects are clarified, planning a peri-operative strategy to support the RV is the next step, with particular attention to which device to use as well as the proper timing if an invasive strategy is planned. Regarding medical therapy, evidence has been gathering on the role of levosimendan as an optimization strategy prior to LVAD, even though conflicting results exist. In cardiac surgery, preoperative administration of levosimendan has proven to be protective against cardiac, renal, and neurological damage in high risk patients [39]. Several studies have demonstrated that levosimendan optimizes RV function by restoring RV-vascular coupling, thus reducing RV pre and afterload as well as increasing RV contractility [40, 41]. Furthermore, Kocabeyoglu et al. showed that patient’s optimization through levosimendan infusion improved impaired end-organ functions and preoperative hemodynamic status in patients awaiting LVAD [42]. However, as mentioned above, so far, no survival benefits have been shown for the use of levosimendan before LVAD patients, mainly because of underpowered studies [43].

Regarding RV mechanical support, if data is more consistent with its use once RHF takes place, its role in preventing it is less established [44]. However, recent evidence suggests that in these patients the early use of temporary RVAD could reduce the mortality rate, as demonstrated by Fisher et al. in 22 patients who underwent a temporary RVAD support within 48 h after LVAD implantation [45]. These results could be explained by an early RVAD implantation with a quick improvement of end-organ perfusion. Furthermore, data encourage the planning of a temporary RVAD placement before LVAD implant, and even if RVAD is unplanned, an early placement rather than a delayed one should be favored [46]. Khorsandi et al. reported higher 30-day survival rate in patients who received temporary RVAD as concurrent with LVAD implant vs. delayed RVAD insertion (93.1% vs. 71.4%) [47]. With regard to the type of device, VA-ECMO or a direct RV support is probably the best choice if RHF occurs during LVAD implant [48]; percutaneous strategies might be advocated due to lower complication rate as well as ensuring early mobilization [49].

Preventive placement of RVAD in LVAD patients with RV dysfunction rather than unplanned RVAD support post-LVAD RV failure has shown to improve outcome. The greatest evidence on mechanical support as preventive measures regards temporary RVADs with or without oxygenator such as ProtekDuo^®^ (TandemLife), Impella RP^®^ (Abiomed), and TandemHeart^®^ (Cardiac Assist, Inc.; Pittsburgh, Pa)[45]. In this regard, recent evidence has been gathered on the placement of ProtekDuo cannula before LVAD implant in patients with borderline RV function in order to avoid the onset of RHF. ProtekDuo cannula is a minimally invasive percutaneous strategy to support the RV, it is a dual-lumen cannula characterized by an outer 29 F lumen and an inner 16 F lumen. It drains venous blood from right atrium to pulmonary trunk, with the possibility to add a pulmonary support through an oxygenator in the circuit. Besides our experience, other studies have showed positive evidence with ProtekDuo [49, 50]. For instance, Schmack et al. showed that ProtekDuo cannula in combination with a TandemHeart pump might combine the advantage of a fully RV support with the minimal invasive percutaneous approach, providing a useful option to avoid RHF, decreasing postoperative complications and allowing for early ventilator weaning and extubation [49]. Salna et al. retrospectively analyzed a cohort of 27 patients who received ProtekDuo RVAD after LVAD implantation, demonstrating that ProtekDuo is safe and feasible and provides high 1-year survival rate in patients with post-LVAD severe acute RHF [50]. Deschka et al. assessed outcomes of a total of 25 LVAD recipients for biventricular impairment who received also unplanned temporary RVAD after a failed attempt to wean from cardiopulmonary bypass due to acute RV failure. Their results encourage the use of peri-operative RVAD in LVAD recipients in presence of biventricular dysfunction since temporary RVAD support enables the RV to adapt to the paraphysiological changes induced by the LVAD [51]. In fact, as mentioned above, once the LV pump is activated, the RV undergoes profound modifications such as increased preload and reduced contractility due to a possible shift of the interventricular septum. That being said, the presence of RVAD gives the RV more time to adapt to these modifications, avoiding the impairment of end-organ perfusion and ultimately RHF. However, future prospective studies are needed to further investigate the role of temporary RVAD in this population, with special attention to timing, type of device, patient’s selection, and the best technique.

In conjunction to mechanical support, it is essential to pursuit hemodynamic optimization as well as inotropic support. In fact, evidence suggests that the longer the duration of RV support and the higher RV flow, the higher the likelihood of complications such as pulmonary bleedings due to continuous blood flow [52]. Therefore, it is of paramount importance to combine the two strategies and carefully select the patients that would benefit the most from preventive measures.

## Conclusions

A thorough evaluation of LVAD candidates is essential to minimize the complication rate and to assure better survival rate. In this context, the risk stratification of LVAD-related RHF is crucial and one of the key elements is preoperative RV function. To date, there are still insufficient data on a proper risk stratification in patients in which RV function simply cannot be classified as either good or unacceptable, the so called “borderline” phenotypes. Therefore, it is necessary to investigate the role of additional strategies to assess the effective RV contractile reserve as well as to develop and validate a universal risk score in a large multicenter patient cohort that focuses on contemporary continuous-flow LVADs. Future research should focus also on the emerging role of temporary RVAD support, especially with regards to the optimal timing to seek this strategy. The overall results will lead to a better prognostic stratification of patients with a borderline RV function as well as further implementation of peri-operative mechanical RV support. The ultimate goal is to face the growing population of advanced heart failure patients and heart donor shortage, allowing to a further rise in LVAD implants and a decrease in complications related to post-operative RV dysfunction.
